# Evaluation of a Multiplex ELISA for Autoantibody Profiling in Patients with Autoimmune Connective Tissue Diseases

**DOI:** 10.1155/2014/896787

**Published:** 2014-01-16

**Authors:** Alejandro Caro Pérez, Sarita Kumble, Krishnanand D. Kumble, M. Consuelo Alonso Cañizal, Luis M. Jiménez Jiménez, Lorena Alonso Díez, Pilar Durán Parejo

**Affiliations:** ^1^Gennova Research, Gennova Scientific S.L. Seville, Spain; ^2^Pictor Limited, Auckland, New Zealand; ^3^Department of Metabolopathy, Hospital Virgen del Rocio, Seville, Spain

## Abstract

The performance of immunoassays for the detection of autoantibodies is of critical importance in the diagnosis and assessment of patients with autoimmune connective tissue diseases (ACTD). Our objective was to compare the features of two multiplexed assays—INNO-LIA ANA and Gennova-PictArray ENA ELISA—for measurement of multiple autoantibodies and their utility as a clinical tool in ACTD diagnosis. The antigens included SS-A/Ro (60 and 52), SSB/La, Sm, Sm/RNP, CENP-B, Jo-1, and Scl-70. Stored sera from 85 ACTD patients and 80 controls consisting of patients with vasculitis, rheumatoid arthritis and infectious diseases, as well as healthy subjects were analyzed jointly with clinical and laboratory data. Agreement between the two methods varied between 58 and 99% (Cohen's kappa: 0.21–0.71) mostly for SSA and SSB. The frequency of specific autoantibodies measured using the two methods was more variable for SSA, SSB, and RNP/Sm. There were a higher number of ambiguous results when using INNO-LIA. The optimized cut-off values of the Gennova-PictArray resulted in over 99% specificities in samples obtained from the control group. Sensitivity patterns were more accurate in Gennova-PictArray than in INNO-LIA, as suggested in previously reported studies. A third method could be applied to determine which of the two methods is more accurate.

## 1. Introduction

The detection of antinuclear antibodies (ANA) has long been an important tool in the early diagnosis of autoimmune connective tissue diseases [1]. The antigens used in their detection are purified by the saline extraction of animal origin nuclei, being termed as extractable nuclear antigens (ENA). The correct use and interpretation of serologic testing for diagnosing autoimmune diseases present a challenge to clinicians for several reasons: (a) the sensitivity and specificity of most laboratory tests for autoimmune disease are significantly less than 100% and (b) detection of autoantibodies using different techniques such as indirect immunofluorescence or multiplex bead assays give different results [2]. Multiplex microarray-based ELISA assays have been reported to provide concordant results when compared with ELISA-based tests [3] with the added advantage offered by multiplexing reduced labor and provision of the complete autoantibody profile with a single test.

Autoantibodies play a role as biomarkers assisting in diagnosis and monitoring of disease activity, predicting disease onset, classifying disease subsets, and defining prognosis [4]. Their detection with high sensitivity and specificity is therefore of the highest importance. Despite various testing methods now being used for autoantibody profiling, new techniques continue to be developed and reported to facilitate diagnosis and therapeutic monitoring in CTD patients [5]. It is critical to evaluate the new methods with those being used in testing laboratories in order to assess their performance as well as to identify deficiencies of methods that are in current use. In this paper, we provide an overview of a new methodology based on ELISA called Gennova-PictArray, in comparison with INNO-LIA based in Western blot immunoassays.

## 2. Materials and Methods

### 2.1. Patients

The study included 85 serum samples submitted to our reference laboratory for autoimmune testing from patients with ACTD. Patients were recruited through input clinical diagnosis according to criteria of SLE diagnosis issued in 1997 by American College of Rheumatology [6]. Samples were tested using Gennova-PictArrays and INNO-LIA and the results used for comparative analysis.

The study also included 80 serum samples from a control group to determine the clinical specificity of both tests. The control group consisted of samples from patients with vasculitis, rheumatoid arthritis, infectious diseases, and healthy subjects. Vasculitis is an accompanying or atypical symptom for a number of rheumatic diseases such as rheumatoid arthritis, systemic lupus erythematosus, and dermatomyositis. Infectious diseases are characterized by a high titre of serum antibodies which may result in false positive results in tests measuring serum antibodies to autoantigens.

### 2.2. Autoantibody Profiling Methods

The most commonly measured ENA specificities are anti-SS-A/Ro (60 and 52), anti-SSB/La, anti-Sm, anti-Sm/RNP, anti-CENP-B, anti-Jo-1, and anti-Scl-70 [7].  Table 1 lists the source and nature of the antigens in the two assay systems being evaluated. Only antigens common in both test kits were compared in this study.

#### 2.2.1. Line Blot Immunoassay (INNO-LIA)

INNO-LIA (Innogenetics NV, Belgium) is a qualitative line blot for identifying the reactivity of serum antibodies to specific antigens coated as discrete lines on a nylon membrane with a plastic backing. Briefly, the strips are incubated in buffer containing blocking protein, followed by incubation with prediluted serum samples. Autoantibodies present in the sample bind to specific antigens deposited on the membrane, which are identified by alkaline phosphatase conjugated anti-human-IgG antibodies showing blue stained bands on the strips. The results are interpreted by comparing the intensity of the reaction with control lines and measured by image analysis. The strips can be scanned and analyzed using software [8]. LIA strips are easy to use, require less processing time, and has a similar sensitivity and specificity to ELISA.

#### 2.2.2. Multiplex Microarray ELISA (Gennova-PictArray)

**Figure 1:**
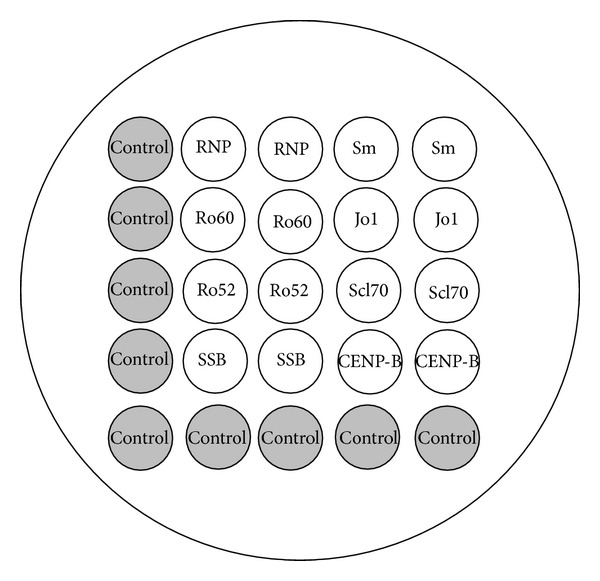
Gennova-PictArray layout for ENA panel including assay controls: RNP/Sm, SSA (Ro60), SSA (Ro52), SSB, Sm Antigen, Jo1, Scl70, and CENP-B.

Microarrays are prepared by depositing 300 *μ*L antigen spots along with a number of control spots included to ensure that the test has been performed correctly. The spots are deposited at the bottom of sixteen specially designed nanowell slides containing a protein binding membrane. The array layout has been shown in Figure 1. After spot deposition, nonspecific binding of serum antibodies to the membrane is reduced by incubation with a protein blocker. The microarray panel consisted of the most commonly detected antigens following positive outcome from ANA testing by immunofluorescence assays.

Diluted serum samples were added to the wells and after incubating at 37°C, the nonbound material was washed followed by sequential incubation with biotin-conjugated anti-human IgG and horseradish peroxidase-conjugated Streptavidin. The amount of IgG bound to antigens was measured by adding diaminobenzidine (DAB) to each well. The peroxidase reaction is stopped by a single wash to remove excess substrate and the wells dried. The colour intensity is proportional to the amount of autoantigens present in the original serum sample. The intensity of spots images was analysed by Pictorial Software which was developed to analyze spot intensities for Gennova-PictArrays.

The method offers the advantages of enabling automated sample processing using off-the-shelf ELISA processors, consistent performance, cost-effectiveness, and precision in measurement of antibody levels.

### 2.3. Interpretation of Results

For INNO-LIA results, are reported as negative (<67% of control line), equivocal (between 67% and 100% of control line), or positive (>100% of control line) according to the manufacturer's recommendations.

Gennova-PictArray results were quantified and reported as IU/mL. This unit reports the fold difference in signal for the patient sample as compared to samples that are negative for the antibodies. The units are interpreted with the following ranges: negative (0–0.8 IU/mL), weak positive (0.9–1.3 IU/mL) or ambiguous result, positive (1.4–25 IU/mL), and strong positive (>25.1 IU/mL).

### 2.4. Statistical Analysis

The analysis was performed using SPSS statistics, version 19.0 (International Business Machines, USA). Agreement between the INNO-LIA and Gennova-PictArrays was assessed using Kappa coefficient as previously described [9, 10]. A recommended interpretation of kappa values is 0.01–0.2 indicating poor agreement, 0.21–0.4 is fair, 0.41–0.6 is moderate, 0.61–0.8 is substantial agreement, and 0.81–0.99 is almost perfect agreement [11].

## 3. Results

### 3.1. Agreement between Autoantibody Assays

The frequency of autoantibodies detected by the two immunoassays is summarized in Table 2. Comparison of the two techniques showed a higher frequency of discrepant results for autoantibodies to SSA, SSB, and RNP/Sm. The rest of the antigens showed concordant results between the two techniques.

There were a higher number of ambiguous results when using INNO-LIA (Table 3). Of all the antigens that gave ambiguous results autoantibodies binding to Ro60 antigen, in serum tested using the INNO-LIA assay resulted in 20 of 85 samples giving ambiguous results, one ambiguous result for Ro60 autoantibody reactivity was obtained with Gennova-PictArray.

As seen in Table 4, the specificity of antibody binding to all antigens in the array but Ro52 was 100% (Ro52 gave a 98.8% specificity).

The agreement between the two assays for autoantibody detection is summarized in Table 5. The observed agreement was consistently ≥90% for anti-RNP/Sm, anti-Sm Antigen, anti-Scl70, and anti-CENP-B. In contrast, the observed agreement for the detection of anti-Ro60, anti-Ro52, and anti-SSB was between 51 and 88%.

The estimated Kappa coefficient for specific agreement between the different assays was “fair” for anti-RNP/Sm, anti-Ro60, and anti-Sm Antigen; “substantial” for anti-Ro52, anti-SSB, anti-Scl70, and anti-CENP-B; and “undefined” for anti-Jo1, due to the high proportion of negative results with INNO-LIA and Gennova-PictArrays.

## 4. Discussion

Quantification of autoantibodies is essential for the diagnosis of autoimmune diseases such as ACTD. Clinicians can use these results as guidance for classifying patients and/or for assessing their response to specific therapies, especially in cases where development of targeted biological therapies is possible [12–14].

There are many factors involved in the measurement of autoantibodies. Patients with ACTD have a high concentration of serum antibodies to autoantigens. New assays need to be developed for measuring newly reported biomarkers for the diagnosis and pathogenesis of this disease [15–17]. Biomarkers can show a different pattern of expression during the evolution of disease [18]. In this variable context, the measurement of a panel of autoantibodies has become an important tool of assessment of ACTD patient status in clinical practice [1, 6].

Recent technologies have provided a new approach for autoantibody quantification based on multiplex testing which represents an advantage compared to earlier methods such as line blot assays and conventional one well-one test microtiter ELISA [5, 19]. These multiplex assays have made it possible to simultaneously detect multiple biomarkers using a single platform and a single serum sample. In this study we have compared two immunoassays for the measurement of autoantibodies to ENA in a cohort of ACTD patients. Our results show different levels of clinical sensitivity for the detection of the most frequently detected autoantibodies in ACTD patients.

Gennova-PictArrays enable the testing of eight antigens in parallel from individual serum samples with high specificity (99.8%). This method provides significant savings in time, avoiding the conventional first screening and post-confirmation algorithm currently being used in most clinical testing laboratories for ACTD sample testing. Gennova-PictArrays require only 50 *μ*L of a 200-fold diluted sample and follows the same steps as a traditional ELISA assay. Together with low setup costs and a fast sample processing time, Gennova-PictArrays provide an affordable alternative to currently available multiplex testing systems. These cost-saving features enable small laboratories with limited budgets to use this technology without a large capital outlay and training of laboratory technicians. Gennova-PictArrays have demonstrated an excellent analytical specificity and sensitivity when compared with established ELISA assays [20]. Moreover, when compared with a line immunoassay, Gennova-PictArray represents the quantitative advantage for clinical management of patients.

The lack of a substantial agreement between techniques and the differences in sensitivities for detection of autoantibodies was relevant in SSA and SSB, the most frequent biomarkers in ACTD according to other studies [21]. This highlights the importance of careful selection of a methodology for the correct diagnostic and consequent classification and treatment of ACTD patients, especially in light of the fact that there is no existence of internationally accepted reference standards for quantification of autoantibodies. It is important to note that differences in antibody binding to antigens can also result from variability in the source and composition of antigens used for autoantibody detection (all but one of the antigens used in INNO-LIA were recombinantly produced while most of the antigens were purified from native sources for Gennova-PictArrays) [20, 22, 23].

Ambiguous results from a diagnostic test leads to uncertain diagnosis and are generally disregarded by clinicians in making treatment decisions. The decrease in the number of ambiguous results by Gennova-PictArray as compared to INNO-LIA assay (36.5% versus 48.2%) may help clinicians make better clinical decisions when using Gennova-PictArray technology.

The results of this study indicate that microarray assays are superior to line immunoassays since selection of assay methodology by a laboratory depends upon several factors such as differences in protocol time, simplicity of use, and cost. It is important to note that clinical service laboratories select tests which can provide results with reliability and confidence to assure correct results, avoiding platforms which require a second test to confirm results [24].

For future analysis of microarray technique, longitudinal cohort studies may be performed which provide new information about ACTD. To determine which method is more accurate, a third method or “positive” reference samples from patients with confirmed Sjogren Syndrome should be tested. This was an exploratory study to assess the use of microarrays in autoimmune diagnostic testing from a clinical angle.

In conclusion, the optimized cut-off values of the Gennova-PictArray resulted in high specificities in samples obtained from healthy donors and other controls (average of 99.8%). Sensitivity patterns were more accurate in Gennova-PictArray than in the line immunoassay, in accordance with the high degree of variability found in other studies. A poor agreement between techniques was observed. The most significant discordance (58–88%) occurred with autoantibodies to SSA and SSB which are the most prevalent specificities identified in ENA profiling and most frequently detected in patients with ACTD [25]. Prospective studies are required to evaluate the significance of a change in level of autoantibodies and to determine the stability of autoantibody profiles over time.

## Figures and Tables

**Table 1:** Source and composition of antigens in INNO-LIA and Gennova-PictArrays.

	INNO-LIA ANA	Gennova-PictArray ENA ELISA
RNP/Sm	Recombinant	Native
Ro60	Native	Native
Ro52	Recombinant	Recombinant
SSB	Recombinant	Native
Jo1	Recombinant	Native
Sm antigen	Modified peptide	Native
Scl70	Recombinant	Native
CENP-B	Recombinant	Recombinant

**Table 2:** The frequency (%) of positive autoantibodies using immunological assays in the connective tissue disease group (*n* = 85).

	INNO-LIA positives (%)	Gennova-PictArray positives (%)
RNP/Sm	1 (0,85)	8 (6,8)
Ro60	12 (10,2)	48 (40,8)
Ro52	37 (31,45)	49 (41,65)
SSB	28 (23,8)	18 (15,3)
Jo1	1 (0,85)	1 (0,85)
Sm antigen	6 (5,1)	2 (1,7)
Scl70	1 (0,85)	2 (1,7)
CENP-B	1 (0,85)	2 (1,7)

Total no. of positives	**87 **	**130**

**Table 3:** The number of ambiguous (+/−) results using immunological assays in the connective tissue disease group.

	INNO-LIA	Gennova-PictArray
RNP/Sm	7	2
Ro60	20	1
Ro52	6	7
SSB	7	9
Jo1	0	2
Sm antigen	1	4
Scl70	0	3
CENP-B	0	3

Total	**41**	**31**

**Table 4:** Prevalence of autoantibodies detected by Gennova-PictArray in different control groups.

	RA (*n* = 7)	V (*n* = 8)	ID (*n* = 35)	HC (*n* = 30)	Total (*n* = 80)
	No. of pos	No. of pos	No. of pos	No. of pos	Specificity
RNP/Sm	0	0	0	0	**100**
Ro60	0	0	0	0	**100**
Ro52	0	0	0	1	**98.8**
SSB	0	0	0	0	**100**
Jo1	0	0	0	0	**100**
Sm antigen	0	0	0	0	**100**
Scl70	0	0	0	0	**100**
CENP-B	0	0	0	0	**100**

				Average (%)	**99.8**

RA: rheumatoid arthritis; V: vasculitis; ID: infectious disease; HC: healthy control.

**Table 5:** Results for connective tissue diseases group. Concordance between INNO-LIA and Gennova-PictArray.

	INNO-LIA compared with Gennova-PictArray	
	Kappa (CI)	Agree (%)
RNP/Sm	0,21 (−0,36–0,77)	92
Ro60	0,22 (0,03–0,42)	58
Ro52	0,63 (0,47–0,79)	81
SSB	0,71 (0,54–0,88)	88
Jo1	not defined	—
Sm Antigen	0,22 (−0,38–0,82)	93
Scl70	0,66 (0–1,32)	99
CENP-B	0,66 (0–1,32)	99

Kappa coefficient (0.01–0.2 indicates poor agreement, 0.21–0.4 is fair, 0.41–0.6 is moderate, and 0.61–0.8 is substantial agreement, 0.81–0.99 is almost perfect agreement).
